# Mass-spectrometry analysis of modifications at DNA termini induced by DNA polymerases

**DOI:** 10.1038/s41598-017-06136-9

**Published:** 2017-07-27

**Authors:** Igor P. Smirnov, Natalia A. Kolganova, Vadim A. Vasiliskov, Alexander V. Chudinov, Edward N. Timofeev

**Affiliations:** 1 W. A. Engelhardt Institute of Molecular Biology Russian Academy of Sciences, Moscow, 119991 Russia; 2 Institute for Physical-Chemical Medicine, Moscow, 119435 Russia

## Abstract

Non-natural nucleotide substrates are widely used in the enzymatic synthesis of modified DNA. The terminal activity of polymerases in the presence of modified nucleotides is an important, but poorly characterized, aspect of enzymatic DNA synthesis. Here, we studied different types of polymerase activity at sequence ends using extendable and non-extendable synthetic models in the presence of the Cy5-dUTP analog Y. In primer extension reactions with selected exonuclease-deficient polymerases, nucleotide Y appeared to be a preferential substrate for non-templated 3′-tailing, as determined by MALDI mass-spectrometry and gel-electrophoresis. This result was further confirmed by the 3′-tailing of a non-extendable hairpin oligonucleotide model. Additionally, DNA polymerases induce an exchange of the 3′ terminal thymidine for a non-natural nucleotide *via* pyrophosphorolysis in the presence of inorganic pyrophosphate. In primer extension reactions, the proofreading polymerases *Vent*, *Pfu*, and *Phusion* did not support the synthesis of Y-modified primer strand. Nevertheless, *Pfu* and *Phusion* polymerases were shown to initiate terminal nucleotide exchange at the template. Unlike non-proofreading polymerases, these two enzymes recruit 3′–5′ exonuclease functions to cleave the 3′ terminal thymidine in the absence of pyrophosphate.

## Introduction

The enzymatic synthesis of chemically modified nucleic acids plays an increasingly important role in life science and biotechnology. Polymerase-assisted incorporation of modified nucleotides has attracted substantial attention over the last decade^1–3^. As a result, many non-natural nucleotides have been comprehensively studied as polymerase substrates. To date, many highly efficient non-natural nucleotide substrates have been proposed. Modified nucleotide analogs have been used in a wide range of applications, including *in vitro* aptamer selection^1, 2^, nucleic acid fluorescent labeling, and biomolecular interaction studies^4, 5^. Furthermore, new artificial DNA polymerases have been evolved that readily incorporate “difficult” nucleotide substrates. A series of crystallographic studies on polymerase complexes with non-natural nucleotides has shed light on the mechanistic aspects of enzymatic synthesis of modified DNA^6, 7^.

Although substantial progress has been made in the field of enzymatic synthesis of modified DNA, certain aspects of polymerase activity in non-natural environments remain unclear. In particular, little or no data are available on the DNA polymerase activity at sequence 3′ termini when using non-natural nucleotides. To a certain extent, this gap in knowledge may be associated with the widespread use of protocols for 3′ DNA tagging. Typically, the needs in the 3′ terminus modifications are satisfied with terminal nucleotidyl transferase tailing or filling 5′ overhangs^8, 9^. Studies on incorporation of modified nucleotides rarely consider the terminal activity of DNA polymerases that is generally associated with proofreading, pyrophosphorolysis, and the synthesis of short 3′ overhangs due to nucleotidyl transferase activity.

With respect to modified substrates, it has been reported that proofreading polymerases are often inefficient at synthesizing full-length, non-natural strands because of their 3′-5′ exonuclease functions^10–13^. This was a major consideration to avoid using proofreading polymerases for incorporating modified substrates or extending over-modified templates. In the context of the terminal activity of the proofreading polymerases, an effect of 3′ nucleotide exchange has been mentioned in a few studies^14–16^. Particularly, dye-labeled dideoxynucleotides R6G-ddCTP and R110-ddUTP were used for terminal nucleotide exchange in the presence of Thermo Sequenase Polymerase and Klenow fragment^16^.

Pyrophosphorolysis is another well-studied phenomenon that may contribute to the modification of sequence termini. It is the reverse reaction of DNA polymerization and proceeds in the presence of inorganic pyrophosphate (PPi), yielding nucleoside triphosphates and shortened DNA strands. It has been demonstrated that the inability of polymerases to extend beyond non-natural bases activates pyrophosphorolysis^17^. The pyrophosphorolysis of non-natural acyclonucleotides, dideoxynucleotides, and other blockers at the 3′ end of the primer has been extensively studied as a key step in pyrophosphorolysis-activated polymerization^18–21^.

Non-templated 3′-tailing of PCR and extension products is probably the most studied terminal activity of polymerases^22, 23^, and it has been widely used for T/A cloning^24–26^. Enzymes that lack proofreading functions are best suited for adding 3′ overhangs. Deoxyadenosine was found to be a preferred natural nucleotide for 3′-tailing. It has been recently shown that a specific modification of the template may prevent 3′-tailing in primer extension (PE)^27^. Although the non-templated addition of natural or modified dNTPs with terminal nucleotide transferase is a routine procedure for DNA tailing at the 3′ end, no attention has been drawn to the transferase activity of commonly used polymerases with respect to modified nucleotide substrates.

In the course of our ongoing studies on development of new Cy3/Cy5 dNTP analogs, we observed two highly specific effects that non-proofreading polymerases induce at the 3′ sequence ends in the presence of a non-natural nucleotide substrate, preferential tailing of DNA with a modified nucleotide and pyrophosphate-dependent terminal nucleotide exchange. In this paper, we describe an effect of Cy5-modified dUTP (Y) on the transferase, pyrophospholytic, and nuclease activities of selected non-proofreading and proofreading DNA polymerases. Nucleotide Y was selected from a series of newly synthesized fluorescent non-natural nucleotide substrates to replace dTTP in PE studies and experiments with short oligonucleotide models. Our data support the preferential tailing of 3′ sequence ends with non-natural nucleotides due to enzyme transferase activity. Another type of modification, nucleotide exchange in the template strand, was a result of pyrophosphorolysis and polymerase activity. We also demonstrate that proofreading polymerases activate the exonuclease function to exchange the 3′ terminal natural nucleotide for Y. The characteristics and extent of modifications were shown to depend on numerous parameters, including the polymerase type, temperature, substrate concentration, and reaction time.

In the current study, MALDI TOF mass-spectrometry was used as a primary analytical technique because of its competence in identifying major reaction products^28^. The commonly used separation of DNA fragments by gel electrophoresis is frequently ineffective in the exact assignment of the product bands. Additionally, modified residues considerably and unpredictably change the mobility of DNA strands in the gel. We demonstrate here that mass-spectrometry is an indispensable tool for analyzing complex enzymatic reactions with relatively short DNA strands.

## Results and Discussion

### Terminal activities of non-proofreading polymerases in the presence of nucleotide analog Y

**Figure 1:**
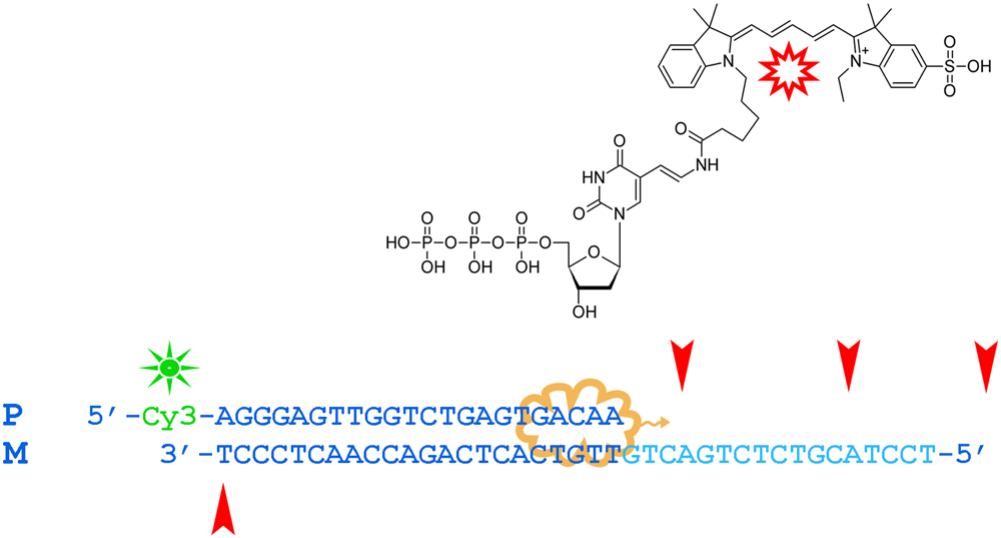
Oligonucleotide model for the PE reaction. The Cy-3′-labeled primer (P) was extended with selected polymerases in the presence of a nucleotide mixture (dATP, dGTP, dCTP, and Y). Red arrows point to sites of nucleotide Y incorporation in the primer or template (M) strands. Cy3 (green) and Cy5 (red) fluorescent labels make detection of the reaction products in the polyacrylamide gel possible *via* two separate channels.

**Figure 2:**
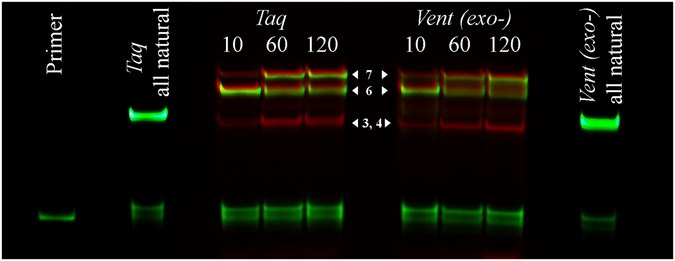
Dual channel overlay image of the gel-electrophoresis of the PE reaction in the presence of *Taq* and *Vent* (exo-) DNA polymerases at 72 °C. PE was performed either with all natural dNTPs for 10 min or using a mixture of dATP, dGTP, dCTP, and Y for varying amounts of time (10, 60, and 120 min). The concentration of the nucleotides was 200 μM (each). Dual-labeled products (red-green bands) with different mobility accumulate in the reaction mixture over the course of the reaction because of Y incorporation. Cy5-labeled strands (red bands) appear over time in the reaction mixture due to modification of the template at the 3′-end. Assignment of bands 3 (M-dT + Y), 4 (M + Y, M-dT + Y + dA, and M-dT + Y + dG), 6 (F), and 7 (F + Y) was made by comparative analysis of electrophoresis and MALDI data.

**Figure 3:**
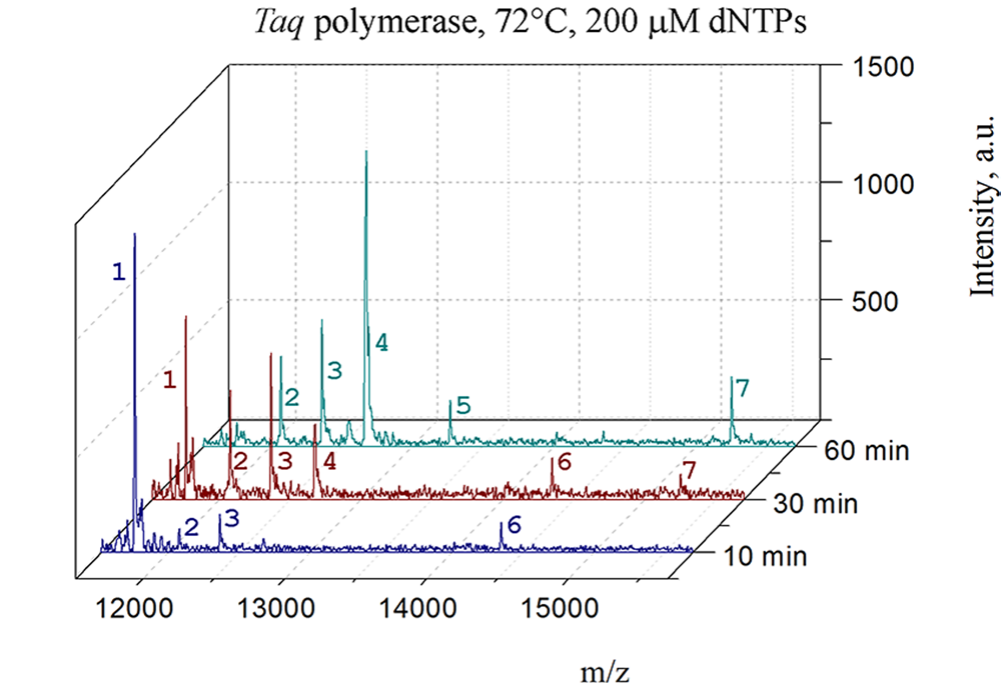
MS analysis of the PE reaction mixtures with *Taq* polymerase at 72 °C. PE was performed with a mixture of dATP, dGTP, dCTP, and Y for varying amounts of time (10, 30, and 60 min). The concentration of the nucleotides was 200 μM (each). The peaks were assigned based on their m/z values as follows: (1) M; (2) M + dA; (3) M-dT + Y; (4) M + Y, M-dT + Y + dA and M-dT + Y + dG; (5) M-dT + Y + Y; (6) F; and (7) F + Y. *Taq* polymerase generates a diverse pool of products because of the enzyme transferase activity and nucleotide exchange via pyrophosphorolysis.

The basic oligonucleotide model for PE that was used in the current study is shown in Fig. 1. It was designed to suit both mass-spectrometry and gel electrophoresis detection by fluorescence. The presence of two different fluorescent tags in the PE products, Cy3 at the primer 5′ end and Cy5 from the incorporation of Y, facilitates the detection and preliminary assignment of bands in the polyacrylamide gel *via* a dual detection channel. The number of modified sites inside the sequence was limited by two non-consecutive incorporations to avoid low-efficiency issues. In our study, we screened five commonly used non-proofreading DNA polymerases (*Taq*, *Vent* (exo-), *DeepVent* (exo-), *Bst 2.0*, and *Therminator*). The color Cy3/Cy5 overlay image of the gel from the PE reactions with *Taq* and *Vent* (exo-) polymerases showed the accumulation of multiple extension products and Cy5-labeled template strands over time (Fig. 2). One major Cy3/Cy5 band at 10 min was observed in the PE reactions for both the *Taq* and *Vent* (exo-) polymerases; it should presumably be assigned to the full-length modified strand (F). Longer exposure generated a mixed Cy3/Cy5 product that has a notably lower mobility. Additionally, one or two Cy5 bands appeared that should be associated with modified template strands. More detailed information is available from the MALDI mass spectra of the reaction mixtures and is shown in Fig. 3 and Suppl Fig. SF1. Extension with *Taq* polymerase results in more diverse reaction mixtures. At 72 °C, we observed that the newly synthesized strand F and template (M) undergo further transformations, yielding F + Y, M + dA, M-dT + Y, M + Y, M-dT + Y + dA, and M-dT + Y + dG strands (Tables 1 and ST1). The latter three products, largely M-dT + Y + dA, constitute peak 4 as their masses fall in the narrow 25-Da range. Typically, the M and F strands were largely depleted after 2 hours of exposure. Additionally, the M-dT + Y + Y strand was generated. Decreasing the temperature to 64 °C or lowering the dNTP concentration slows down the terminal transformations, although it yields the same pool of products. The role of temperature in the rate and extent of terminal modifications is likely related to the enzyme working optimum rather than the thermal stability of DNA duplex. The *Taq* DNA polymerase has an optimal temperature approximately 72 °C, which is typically higher than the T_m_ value of the primer/template duplex in most applications. In the present PE model, the T_m_value of the full-length duplex M:F in the reaction buffer is 68 °C. Therefore, partial duplex unwinding at sequence ends is expected at either temperature (72 or 64 °C). In the PE reaction with *Vent* (exo-) polymerase, only two major modification products were observed at 72 °C: F + Y and M-dT + Y. At a lower temperature, an additional strand, F + dA, was observed.

**Table 1:** Products of polymerase-assisted terminal modification reactions with extendable and non-extendable models.

Model	Polymerase	dNTPs	T, °C	Reaction product (MS peak)
M:P	Taq	d(AGC), Y	72/64	M + dA (2), M-dT + Y (3), M + Y (4), M-dT + Y + dA (4), M-dT + Y + dG (4), M-dT + Y + Y (5), F (6) F + Y (7)
M:P	*Vent (exo-)*	d(AGC), Y	72/64	F + Y (7), M-dT + Y (3), F + dA (9)
M:P	*D.Vent (exo-)*	d(AGC), Y	72	F (6), M + dA (2), M-dT + Y (3)
M:P	*Bst 2.0*	d(AGC), Y	72	F (6)
M:P	*Therminator*	d(AGC), Y	72	M + dA/dG (2), M + dA + dA (10), M + Y (4), M-dT + Y + dA (4), M + Y + dA (11), M-dT + Y + Y (5)
M:P	*Pfu*	d(AGC), Y	72	M-dT + Y (3)
M:P	*Phusion*	d(AGC), Y	72	M-dT + Y (3), P + CAGYCAGAGACGY (8)
H1	*Taq*	d(AGC)	64	H1 + dA
H1	*Taq*	Y	64	H1 + Y
H1	*Taq*	d(AGC), Y	64	H1 + Y
H1/PPi	*Taq*	Y	64	H1-dT + Y
H4/PPi	*Taq*	Y	64	H4-dT + Y
H4	*Therminator*	Y	64	H4 + Y
H4/PPi	*Therminator*	Y	64	H4-dT + Y + Y
H4	*Pfu*	Y	64	H4-dT
H4	*Phusion*	Y	64	H4-dT + Y
H4/PPi	*Pfu*	Y	64	H4-dT + Y
H4/PPi	*Phusion*	Y	64	H4-dT + Y

In the presence of hydrophobic Cy5-dUTP, the ability of the *Taq* and *Vent* (exo-) polymerases to add non-templated overhangs at the 3′ end of the F strand almost completely turned toward the non-natural nucleotide. The incorporation of the non-templated base by polymerases in many aspects resembles translesion DNA synthesis through an abasic site, which is probably the most frequent and significant type of DNA damage^29^. Importantly, there is a lack of a Watson-Crick counterpart in either case. Generally, translesion synthesis may also be considered non-templated. Moreover, most polymerases comply with the “A-rule” when adding an overhang or performing the synthesis opposite an abasic site^30–33^. The structural basis for the “A rule” was established by NMR and thermodynamic studies that proposed adenosine stacking within the helix when placed opposite an abasic site in a duplex^34, 35^. Detailed studies with the non-natural hydrophobic dNTP analogs, pyrene and 5-nitroindole, in translesion DNA synthesis showed that these non-natural nucleotides incorporate much more efficiently than dATP^36^. We observed the same preference for the non-natural nucleotide Y over dATP when adding overhangs with the *Taq* and *Vent* (exo-) polymerases. This concurrence for the two types of polymerase activity argue for very similar mechanisms that may essentially involve hydrophobic and stacking interactions between the incoming dNTP, terminal base pair, and polymerase active center. An exclusive role for hydrophobic interactions is supported by the fact that polymerases from different families share high affinities for non-natural substrates.

Modification of the template M suggests an important role of pyrophosphorolysis, which is the only proofreading activity found in *exo-* polymerases. The formation of M-dT + Y was the primary template modification in the PE reactions with *Vent* (exo-) polymerase, while *Taq* polymerase further converts this product into M-dT + Y + dA, M-dT + Y + dG and M-dT + Y + Y. The prevalence of pyrophosphorolysis over polymerization results from inability of the polymerase to proceed. It has previously been shown that failure to form a successful ternary complex with any of the present nucleotide substrates reverses polymerization^17, 37, 38^. This may occur for non-cognate nucleotide substrates, non-natural bases in the template, or at the sequence ends. Excision of the 3′ base by pyrophosphorolysis is followed by rapid extension in the presence of the most suitable nucleotide. In our experimental setup, this is always the 2′-deoxyuridine analog Y. The excision of the thymidine in M resumes polymerization with the thymidine analog Y. At this point, the *Vent* (exo-) polymerase stops and *Taq* polymerase continues adding dA, dG, or a Y overhang. The presence of the fluorescent Cy3 tag at the 5′ end of the primer may arguably influence template base excision. Nevertheless, we also observed excision of the 3′ terminal dT and base exchange in the template when an unmodified primer was used in the PE reaction (Suppl Fig. SF2). It is worth noting that the presence of modification at the primer 5′ end plays an important role in directing non-templated base addition by *Taq* polymerase. At the template 3′ end, dA was added more efficiently than Y, as seen in Fig. 3 (peaks 2 and 4). Accordingly, this reversal effect has not been observed in the PE reaction with unmodified primer (Fig. SF2).

**Figure 4:**
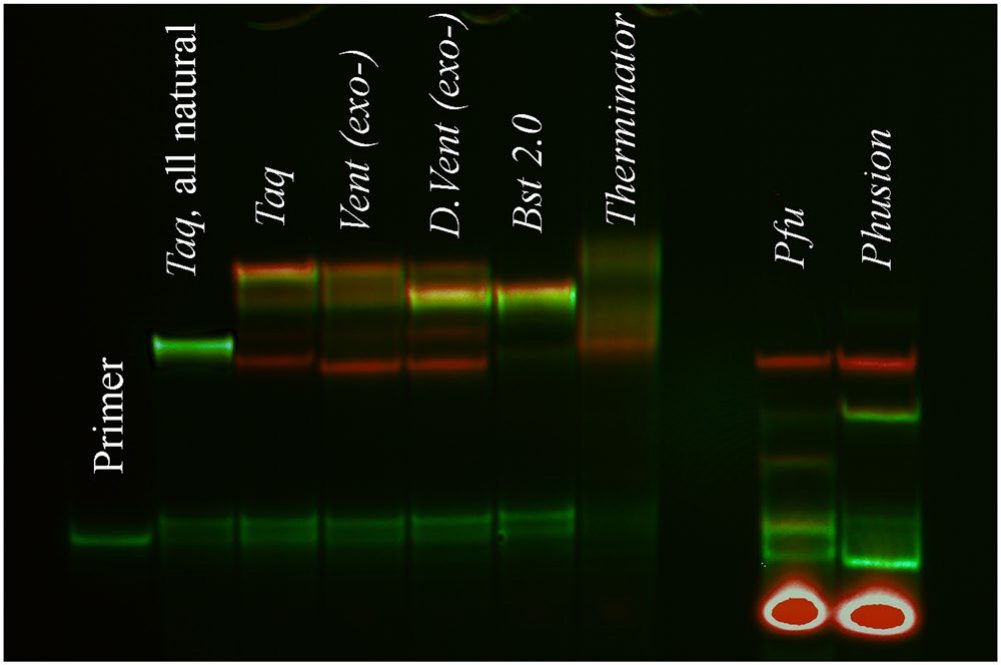
Gel-electrophoresis of the PE reactions in the presence of different exonuclease-deficient and proofreading DNA polymerases at 72 °C. The reaction was performed for 2 h at dNTP concentrations of 200 μM. The ability to modify the sequence ends decreases in the following order for non-proofreading polymerases: *Taq* > *Vent* (exo-) > *DeepVent* (exo-) > *Bst 2.0*. The *Therminator* polymerase yields poor primer extension. The proofreading polymerases *Pfu* and *Phusion* can modify the M strand via terminal dT/Y exchange.

The use of the *DeepVent* (exo-) polymerase in the PE reaction considerably minimized modification of the F strand (Suppl Fig. SF3), which was complemented by only a minor F + dA peak, as detected by MALDI analysis. Template modification was also substantially decreased. Similar to reactions with the *Taq* and *Vent* (exo-) polymerases, extension with *DeepVent* (exo-) also yielded M-dT + Y. However, a large fraction of the template strand remained unaffected. The strand modifications after 2 h of PE at 72 °C were also minimized when utilizing *Bst 2.0* DNA polymerase. Gel electrophoresis showed the absence of modified template M, while uncapped strand F was a major constituent of the reaction mixture (Fig. 4). This result was further confirmed by MALDI analysis (Suppl Fig. SF4). One possible reason for such a “clean” polymerization may be the thermal deactivation of the polymerase itself. The optimum temperature for *Bst 2.0* is 65 °C, while deactivation takes 5–20 min at 80 °C. Modifications at the sequence termini may have been limited by the short enzyme lifetime. On the other hand, the intrinsic ability of *Bst 2.0* polymerase to avoid 3′ end modifications cannot be ruled out.

Finally, we added the *Therminator* DNA polymerase to the study of non-proofreading enzymes. This polymerase is known for its enhanced ability to incorporate certain non-natural nucleotides^39, 40^. Despite this ability, both gel electrophoresis and MALDI analysis revealed poor extension of the primer with Cy5-labeled dUTP. Only low-intensity smeared bands were visible in the gel in the regions of newly synthesized strand and modified template (Fig. 4). MALDI analysis shows the formation of multiple template modification products and an enhanced ability to add single or two-base overhangs to the M and M-dT strands (Suppl Fig. SF5). The latter feature was previously reported for the *Therminator* DNA polymerase^41^. No F strand variants were detected by MALDI mass spectrometry for this polymerase.

### Studies on 3′-tailing and nucleotide exchange with non-extendable hairpin models

**Figure 5:**
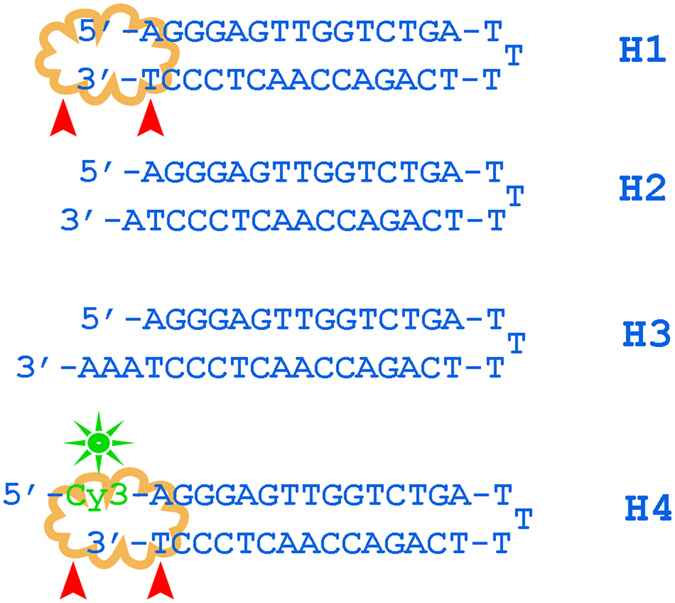
Oligonucleotide hairpin models for studying 3′-tailing and terminal nucleotide exchange: blunt-ended hairpin H1, single dA 3′-overhang in H2, triple dA 3′-overhang in H3, and 5′-Cy3 labeled hairpin H4.

**Figure 6:**
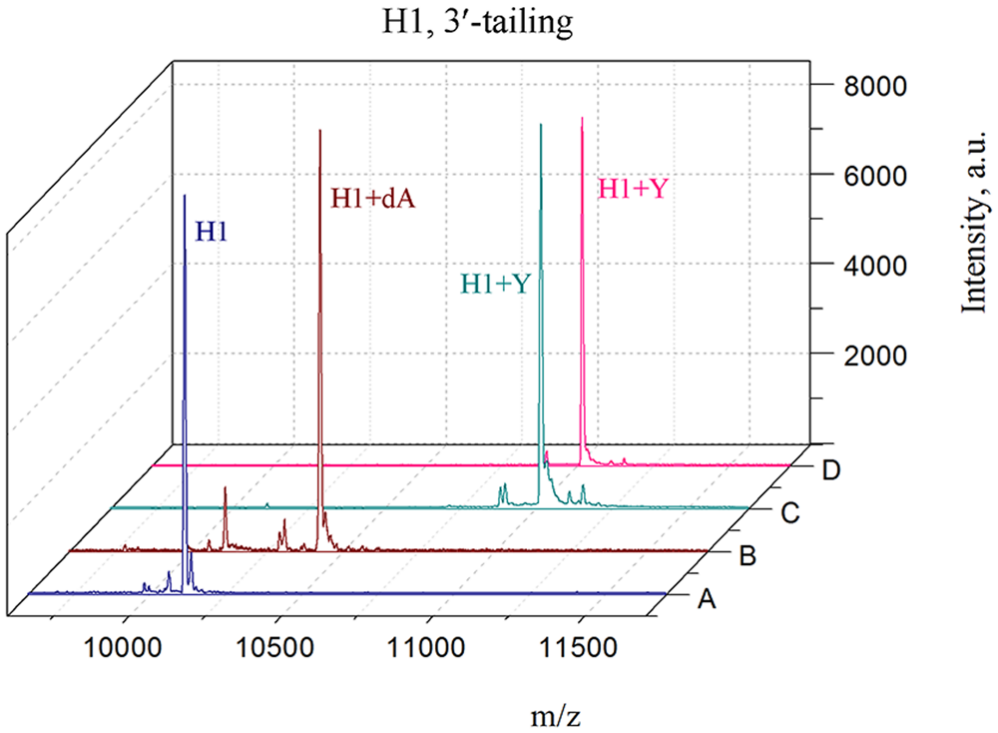
MS analysis of the tailing reaction with *Taq* polymerase using oligonucleotide hairpin H1. (**A**) Reference oligonucleotide H1. (**B**) Tailing reaction at 64 °C for 2 hours in the presence of 200 μM natural dNTPs (dA, dG, and dC). (**C**) Tailing reaction in the presence of Y alone. (**D**) Exposure of H1 to *Taq* DNA polymerase in the presence of the nucleotide mixture dA, dG, dC, and Y. Non-natural nucleotide Y has been demonstrated to be a preferred substrate in the 3′-tailing reaction.

Pyrophosphorolysis of the terminal base in M is possible due to the presence of inorganic PPi, which arises as a by-product of the PE reaction. In contrast, adding the 3′ overhang does not require the presence of PPi. To differentiate between 3′ end tailing with non-natural nucleotide and terminal dT/Y exchange, we further used non-extendable synthetic hairpin models (Fig. 5). The ability of the nucleotide Y to successfully compete against dA in the 3′ transferase reaction was studied with unlabeled hairpins H1-H3 and Taq DNA polymerase in the absence of exogenous PPi. Three 15-bp duplex hairpins were designed to have either a blunt end (H1) or one or three dA overhangs (H2 and H3, respectively). Predictably, exposure of a DNA duplex bearing one or three dA overhangs to polymerase in the presence of different dNTP combinations did not result in extra base addition. The blunt-ended duplex was extended by a single dA in the presence of three natural dNTPs (dA, dG, and dC). Incubation of hairpin H1 with Y resulted in complete conversion of H1 to the H1 + Y strand. When a mixture of Y, dA, dG, and dC nucleotides was used in the tailing reaction, only the H1 + Y strand could be observed as a single overhang product (Fig. 6). These observations fully agree with the results from the PE studies and confirm that the hydrophobic Cy5 deoxyuridine analog Y is a preferred substrate for the non-templated 3′-tailing reaction.

Terminal base excision by pyrophosphorolysis and replacement of the dT with Y at the 3′ end of hairpin H1 was induced by PPi at a concentration of 0.4 mM. The modified strand, H1-dT + Y, was a major reaction product after 2 h of hairpin exposure to *Taq* polymerase in the presence of Y and PPi (Suppl Fig. SF6). Similarly, Cy3-tagged hairpin H4 produced H4-dT + Y strand in the presence of PPi. At a PPi concentration of 0.08 mM, the efficiency of the terminal nucleotide exchange in H1 was somewhat lower than that at 0.4 mM. On the other hand, excessive PPi was reported to inhibit pyrophosphorolysis^38^ due to depletion of Mg^2+^, which is required for catalysis^42^. We also observed this effect when adding PPi up to 1.2 mM.

The explicit ability of the *Therminator* polymerase to add overhangs was confirmed in the tailing and nucleotide exchange experiments with Cy3-labeled hairpin H4. We observed a complete conversion of H4 to H4 + Y in the absence of PPi. The addition of PPi at 0.4 mM induced the formation of H4-dT + Y + Y as virtually the only reaction product.

Our results with the non-extendable hairpin models imply that the presence of PPi and its concentration are determining factors for the type of polymerase activity at the DNA blunt ends until the latter is accessible. In this way, PPi-dependent terminal nucleotide exchange may be initiated if the modified nucleotide analog is the only available cognate substrate. Building 3′ overhangs supposedly terminates catalytic activity of non-proofreading polymerases at that respective strand. The ability of selected non-natural nucleotides to serve as preferential substrates in 3′-tailing may have important implications for DNA labeling and bioconjugation with commonly used polymerases.

### Terminal activity of proofreading polymerases

**Figure 7:**
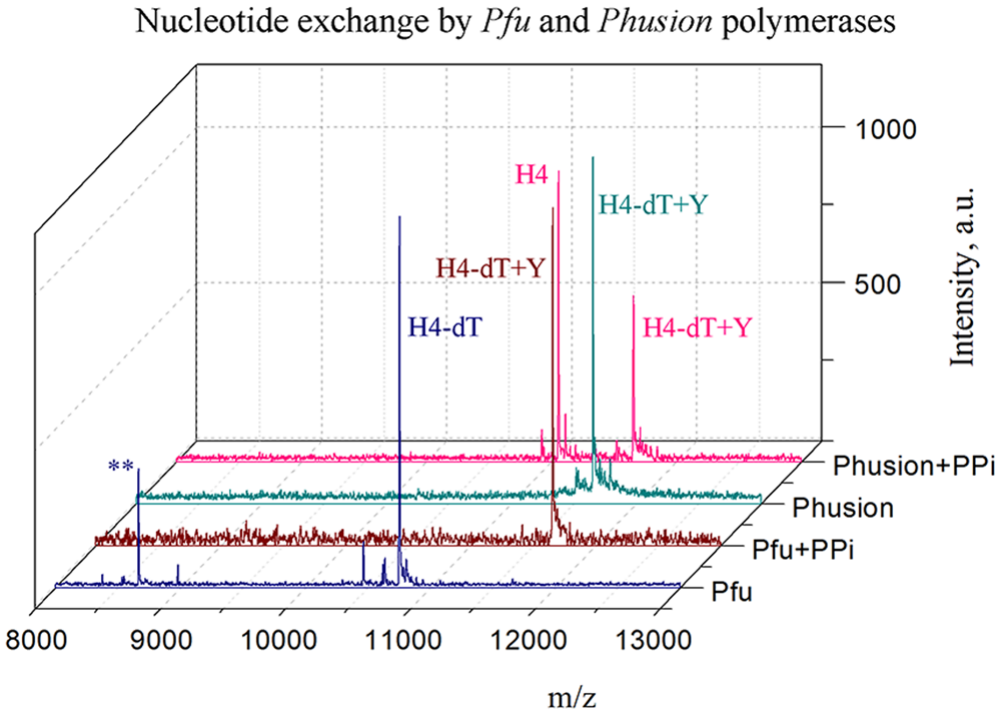
MS analysis of the hairpin modification (H4) by the *Phusion* and *Pfu* polymerases at 64 °C for 2 hours in the presence of 200 μM nucleotide Y. *Pfu* polymerase can excise the terminal dT in the absence of PPi. Partially degraded hairpin Cy3-AGGGAGTTGGTCTGATT-TTCAGACC (**) was also detected in the spectrum. Nucleotide exchange was only observed in the presence of 0.4 mM PPi. *Phusion* polymerase can generate the H4-dT + Y product without PPi because of exonuclease and polymerase activities.

Exonuclease-proficient DNA polymerases have been reported to degrade primers when attempting to extend with non-natural nucleotides or using the non-natural template strand^10, 11^. Similarly, in the PE reactions, we failed to observe full-sized modified strands with the *Pfu, Phusion* and *Vent* polymerases. However, these polymerases displayed markedly different proofreading activities. *Vent* polymerase induced deep degradation of the primer. *Pfu* yielded traces of partially synthesized modified strand and degraded primer. *Phusion* polymerase produced a truncated modified strand that was identified from the mass spectrum of the reaction mixture (Fig. 4 and Suppl Fig. SF7). Polymerization was stalled immediately after the second incorporation of the modified nucleotide. These results agree with previous reports on proofreading polymerases as far as they concern the newly synthesized modified strand. More interesting is that the proofreading polymerases *Pfu* and *Phusion* were able to modify the template strand without degrading it. Importantly, this effect was observed in the absence of the second non-proofreading polymerase, which is unlike previously reported terminal exchange for dye-labeled dideoxynucleotides^16^. We found that these two polymerases induced 3′ terminal nucleotide exchange dT/Y in the template strand M, which implies pyrophosphorolysis or exonuclease excision of the terminal nucleotide. The respective product (M-dT + Y) was found in the mass spectra and gel-electrophoresis. Additional details were obtained from the non-extendable hairpin model H4. When exposed to these two enzymes, the labeled hairpin H4 undergoes terminal nucleotide excision in the absence of PPi (Fig. 7). *Pfu* polymerase promoted nucleotide exchange only in the presence of PPi, while *Phusion* could catalyze this process in the absence of PPi. Without exogenous PPi, *Pfu* polymerase can excise either a single terminal nucleotide or eight nucleotides at the 3′ end. To proceed with exonucleolytic cleavage, the 3′ end of the hairpin has to originally be bound at or translocated into exonuclease domain^43–45^. In this case, *Pfu* polymerase generates partially degraded hairpin along with H4-dT. However, when PPi is added, no degraded product is observed, suggesting that single nucleotide cleavage may proceed in the polymerization domain *via* a reverse reaction. This assumption implies that binding of PPi prevents translocation of the 3′ end strand to the exonuclease domain. Our data do not differentiate between exonuclease cleavage and pyrophosphorolysis in the presence of PPi. Nevertheless, the observation that the presence of PPi was necessary for nucleotide exchange by *Pfu* polymerase argues for an active role for pyrophosphorolysis in the case. These results provide a direct indication that proofreading polymerases can recruit both pyrophosphorolysis and exonuclease activity at the unmodified strand to promote terminal nucleotide exchange.

*Phusion* DNA polymerase is a *Pfu*-based DNA polymerase that has been attached to the nonspecific dsDNA binding protein Sso7d^46, 47^. An additional DNA binding domain in *Phusion* polymerase may account for the observed difference between these two enzymes.

Our findings provide a basis for the modification of DNA sequence termini using selected proofreading DNA polymerases. Given their inability to support the enzymatic synthesis of the modified strand, these polymerases possess an attractive potential to modify DNA strands by terminal nucleotide exchange. Unlike the preferential addition of specific nucleotides by 3′-tailing, this reaction relies on base-pairing rules and is expected to support a wider range of modified substrates for binding functional tags.

## Conclusions

In this paper, we evaluated an effect of non-natural nucleotide on the catalytic activity of DNA polymerases at sequence ends. When binding blunt-ended DNA, polymerases initiate non-templated base addition, pyrophosphorolysis or exonuclease cleavage. None of these processes is dependent on prior polymerization and any of them may proceed after the polymerase has completed new strand synthesis and dissociated. In the presence of modified nucleotide substrate Y, the non-proofreading polymerases *Taq* and *Vent* (exo-) preferentially add non-natural extra base at the 3′ end of the newly synthesized strand. This ability is notably suppressed for the *DeepVent* (exo-) and *Bst 2.0* polymerases. When PPi is present in the reaction mixture, exonuclease-deficient polymerases replace terminal dT with Y, which is the only available thymidine analog, *via* pyrophosphorolysis. The *Taq* and *Therminator* polymerases were shown to additionally activate transferase function after nucleotide exchange. The proofreading polymerases *Pfu* and *Phusion* also initiate 3′-nucleotide exchange. However, this event may be triggered in the absence of PPi due to exonuclease activity. These details provide important insights into polymerase activities at DNA termini in native and non-natural environments. From the practical perspective, our results may be useful for a broad range of applications related to DNA modification.

## Methods

### Oligonucleotides

DNA oligomers were synthesized using an ABI 3400 DNA/RNA synthesizer and purified by reverse phase HPLC. The amino-modifier for labeling the 5′-end of the primer was synthesized from 5′*O*-dimethoxytrityl thymidine by selective alkylation at *N*3 as described in the Supplementary Information. Primer labeling was performed using activated Cy3 dye^48^ following a published protocol^49^. The compositions of all synthesized oligonucleotides were confirmed by MALDI mass spectrometry analysis.

### Synthesis of Cy5-dUTP (Y)

Modified 2′-deoxyuridine triphosphate was synthesized by coupling 5-(3-aminoallyl)-2′-deoxyuridine 5′-triphosphate (Biosan, Russia) with activated Cy5 dye. Asymmetric Cy5 dye was prepared according to published procedures with modifications^50–54^. The details of the synthetic procedure are given in the Supplementary Information.

### Primer extension and hairpin modification

DNA polymerases were purchased from New England Biolabs (*Vent*, *Vent* (exo-), *DeepVent* (exo-), *Bst 2.0*, and *Therminator*) and Thermo Scientific (*Taq*, *Pfu*, and *Phusion*). The reaction mixture (25 μL) contained either 4 μM primer and template or 4 μM hairpin oligonucleotides. The dNTP concentrations were 40 or 200 μM (each) in corresponding buffer. ThermoPol^®^ buffer (New England Biolabs) was used for the *Vent*, *Vent* (exo-), *DeepVent* (exo-), *Bst 2.0*, and *Therminator* polymerases. A buffer supplied by the manufacturers was used in the reactions with *Taq*, *Pfu*, and *Phusion* polymerases. One and a half microliters of 25 mM MgCl_2_were added to the reaction mixtures with *Taq* polymerase. Two units of enzyme were used for all polymerases, except *Bst2.0* (8 units). To initiate pyrophosphorolysis, PPi was added to the reaction mixture at 0.4 mM if not otherwise stated. The reaction mixtures were heated to 95 °C for 1 min and subsequently cooled to 55 °C for 1 min. PE or hairpin modification reactions were performed at 64 or 72 °C for varying amounts of time.

### Polyacrylamide gel electrophoresis

Strand separation after the PE reactions was performed on a 20% polyacrylamide (19:1) gel containing 1xTBE and 8 M urea. Electrophoresis was performed using 1xTBE as the running buffer at 45 °C and 30 V/cm. PE reaction mixtures were precipitated with 2% LiClO_4_ in acetone and diluted in 10 μL of 8 M urea in D_2_O. Before loading onto the gel, the samples were heated for 1 min at 95 °C. The fluorescent bands were separately visualized in Cy3 and Cy5 channels using a research custom-made imaging system.

### MALDI analysis

Mass spectrometry analysis was performed with a 4800 Plus (Sciex) or Microflex (Bruker Daltonics) MALDI-TOF mass spectrometer using 0.25 M aqueous 3-hydroxypicolinic acid as the matrix and 10 mM diammonium citrate as an additive. Spectra were recorded for positive ions. Before analysis, the samples were desalted and concentrated using ZipTip C18 micropipette tips (Millipore).

## Electronic supplementary material

Supplementary Information
