# Supplementary material for: Mass-spectrometry analysis of modifications at DNA termini induced by DNA polymerases
Source: Sci Rep. 2017 Jul 27;7:6674. doi: 10.1038/s41598-017-06136-9 (PMC5532294; doi:10.1038/s41598-017-06136-9)
Supplement: Supplementary file 1 — Supplementary Information [file 41598_2017_6136_MOESM1_ESM.pdf]

Mass-spectrometry analysis of modifications at DNA termini induced by DNA polymerases

Igor P. Smirnov*[2], Natalia A. Kolganova[1], Vadim A. Vasiliskov[1], Alexander V. Chudinov[1], Edward N. Timofeev*[1].

[1]W. A. Engelhardt Institute of Molecular Biology Russian Academy of Sciences, Moscow 119991, Russia. [2]Institute for Physical-Chemical Medicine, Moscow 119435, Russia. *These authors contributed equally to this work. Correspondence and requests for materials should be addressed to E.N.T. (email: edward@eimb.ru)

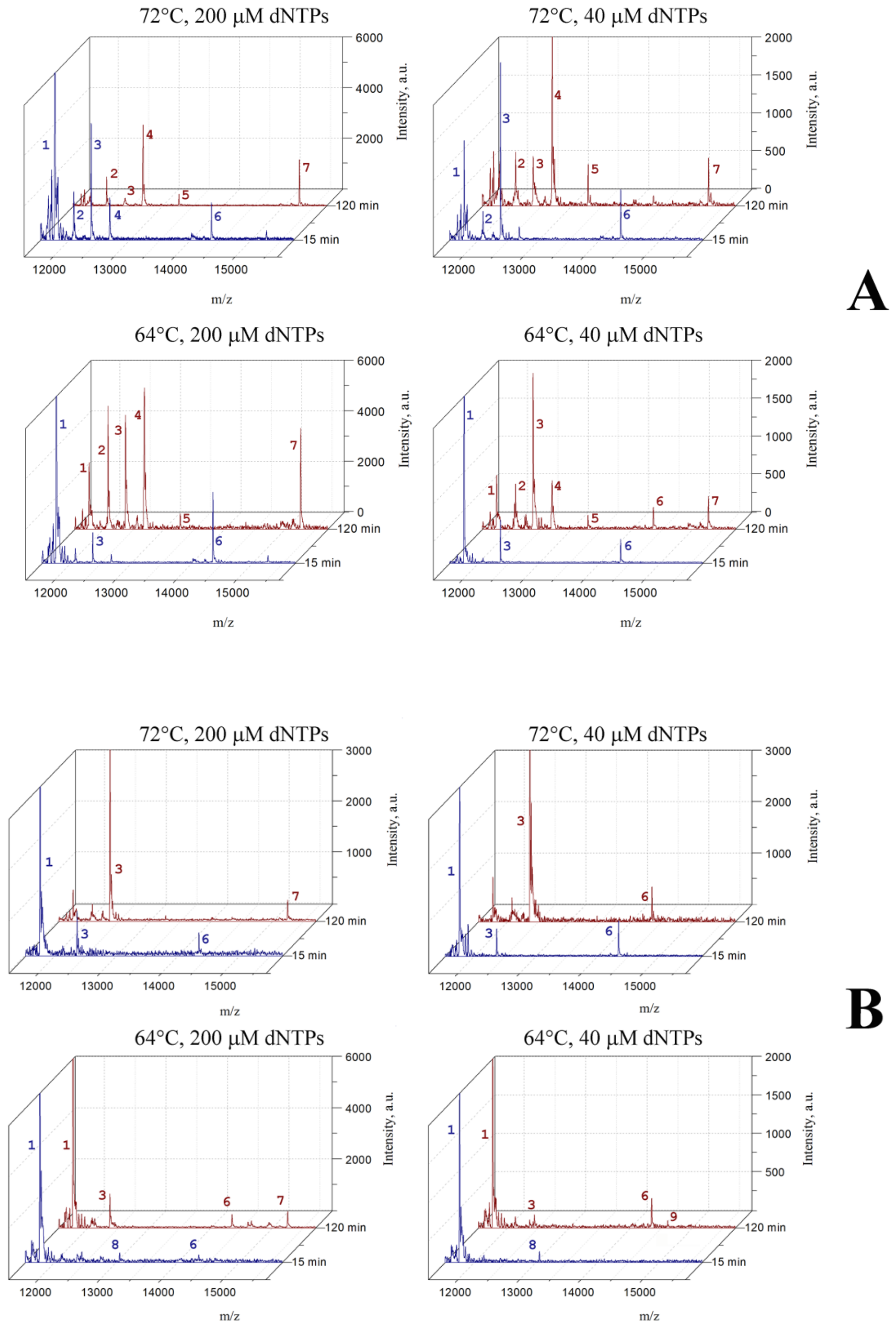

Figure SF1. MALDI TOF MS analysis of the PE reaction mixtures with (A) *Taq* and (B) *Vent* (exo-) polymerases. Two different temperatures (72 and 64°C) were used in the PE reactions. The dNTP concentrations were 200 or 40 μM (each). Terminal activity of the *Taq* polymerase yields a more diverse pool of products. The Vent (exo-) major reaction products are M-dT+Y, F, and F+Y. Higher temperature or dNTP concentration accelerates the accumulation of modified strands. Peak labels: (1) M; (2) M+dA; (3) M-dT+Y; (4) M+Y, M-dT+Y+dA and M-dT+Y+dG; (5) M-dT+Y+Y; (6) F; (7) F+Y; (8) incomplete extension, P+CAGYCAGAGACGY; (9) F+dA.

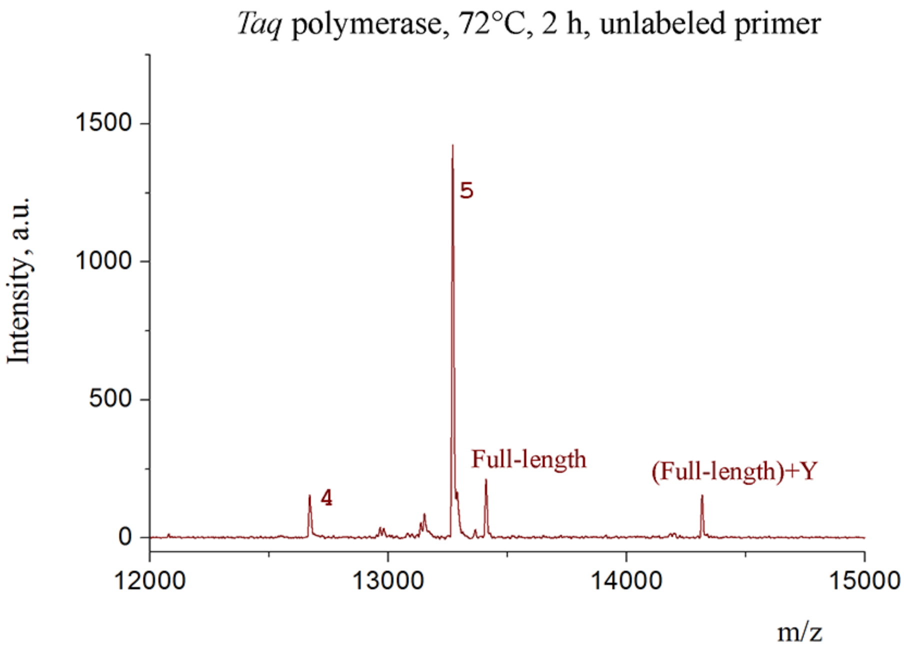

Figure SF2. Mass-spectrum of the PE reaction mixture with *Taq* polymerase using unlabeled primer at 72°C and 200 μM nucleotide concentration. The template modification products (4) M+Y, M-dT+Y+dA, and M-dT+Y+dG, and (5) M-dT+Y+Y were formed as a result of initial nucleotide excision at the 3'-end by pyrophosphorolysis.

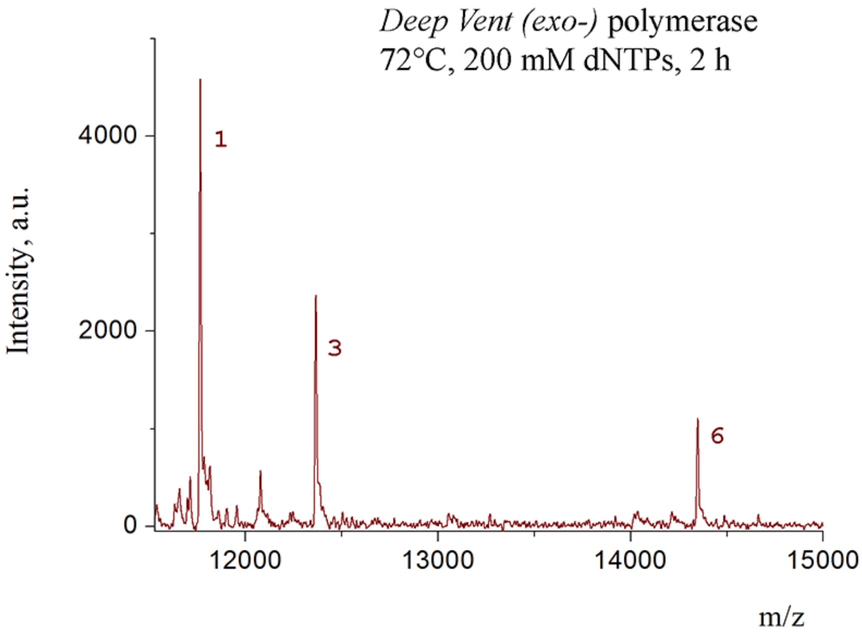

Figure SF3. MALDI mass-spectrum of the PE reaction mixture with *DeepVent* (exo-) polymerase at 72°C and 200 μM nucleotide concentration. The ability to add 3'-overhangs at strand F is virtually suppressed. Template modification proceeds with reduced efficiency yielding M-dT+Y (peak 3).

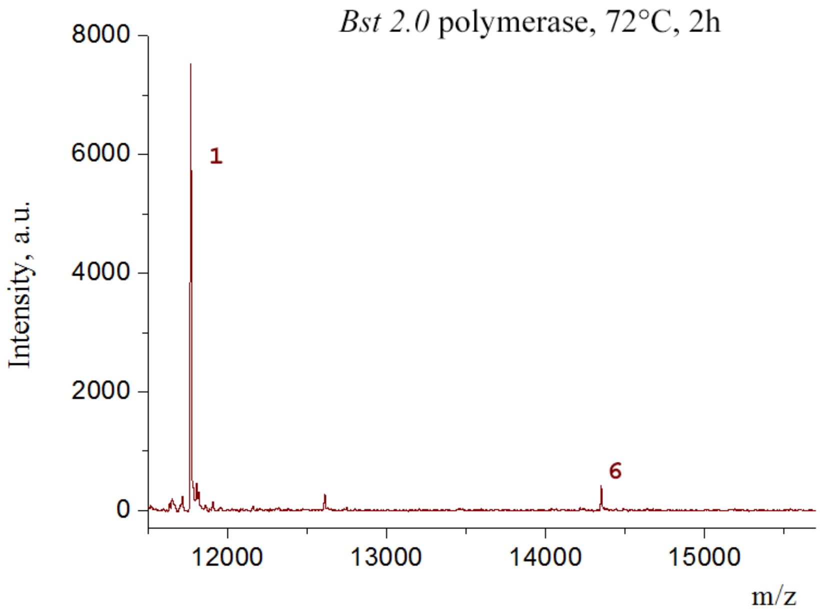

Figure SF4. Mass-spectrum of the PE reaction mixture with *Bst 2.0* polymerase at 72°C and 200 μM nucleotide concentration. Full-size strand F (peak 6) was the major reaction product.

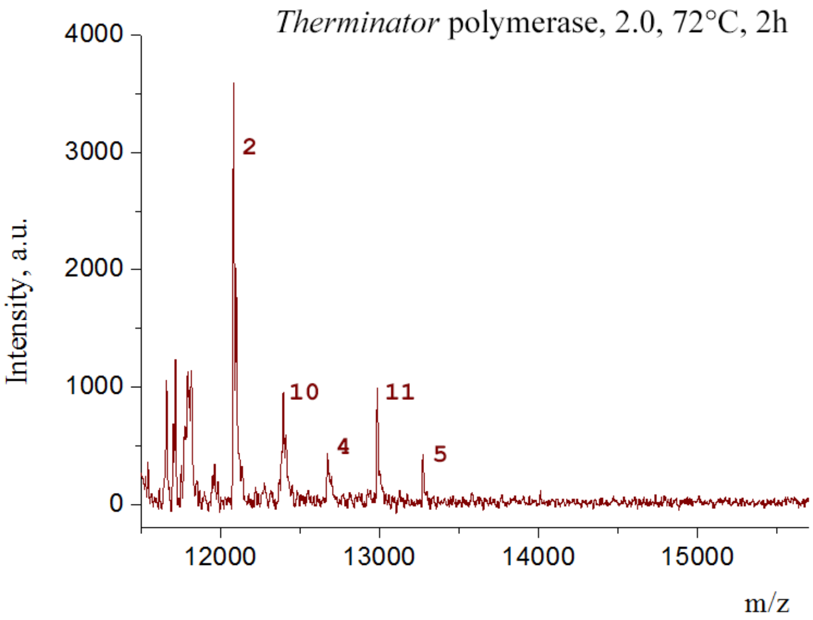

Figure SF5. Mass-spectrum of the PE reaction mixture with *Therminator* DNA polymerase at 72°C and 200 μM nucleotide concentration. MALDI analysis evidences formation of multiple template modification products: (2) M+dA, (4) M+Y, M-dT+Y+dA, and M-dT+Y+dG, (5) M-dT+Y+Y, (10)), M+dA+dA, (11) M+Y+dA.

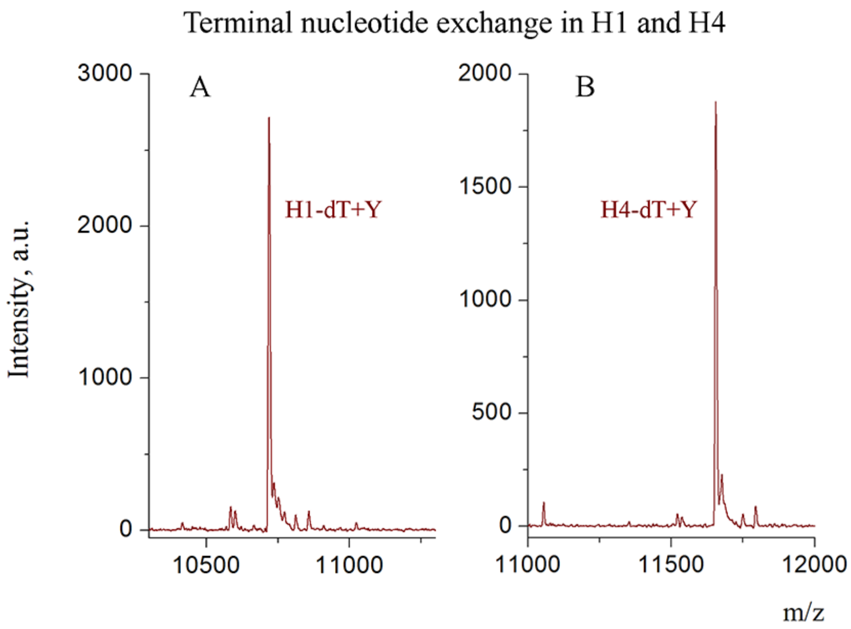

Figure SF6. MS analysis of pyrophosphate-induced terminal nucleotide exchange in hairpin models with *Taq* DNA polymerase. (A) Non-labeled blunt-ended hairpin H1 in the presence of 200 μM nucleotide Y (2 h exposure at 64°C). (B) The same reaction with Cy3-labeled hairpin H4. The modified strand (H1-dT+Y or H4-dT+Y) was a major reaction product in both cases.

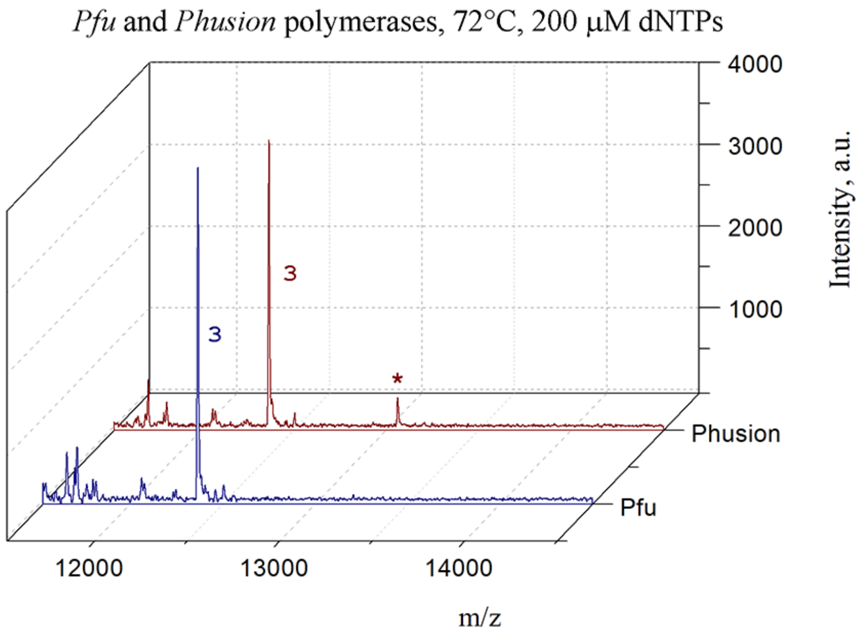

Figure SF7. MALDI mass-spectra of the PE reaction mixtures with the *Phusion* and *Pfu* polymerases at 72°C and 200 μM nucleotide concentration. Proofreading polymerases do not support the synthesis of the modified strand. Only the truncated primer strand P + CAGYCAGAGACGY (*) was detected in the reaction with *Phusion* DNA polymerase. The modified template strand M-dT+Y (peak 3) was detected as a product of the nucleotide exchange reaction.

Table ST1. Oligonucleotides and reaction products.

Oligonucleotide	Length, nt.	Sequence	$[M+H]^+$, calcd.*
Unlabeled primer	22	AGGGAGTTGGTCTGAGTGACAA	6880.50
Cy3-Primer, P	23	Cy3-AGGGAGTTGGTCTGAGTGACAA	7817.53
Template, M	39	TCCTACGTCTCTGACTGTTGTCACTCAGACCAACTCCCT	11765.54
Hairpin H1	33	AGGGAGTTGGTCTGATTTTCAGACCAACTCCCT	10120.53
Hairpin H2	34	AGGGAGTTGGTCTGATTTTCAGACCAACTCCCTA	10433.74
Hairpin H3	36	AGGGAGTTGGTCTGATTTTCAGACCAACTCCCTAAA	11060.16
Hairpin H4 (Cy3)	34	Cy3-AGGGAGTTGGTCTGATTTTCAGACCAACTCCCT	11057.56
Extension product with unlabeled primer	39	AGGGAGTTGGTCTGAGTGACAACAGYCAGAGACGYAGGA	13412.52
Extension product with unlabeled primer+Y	40	AGGGAGTTGGTCTGAGTGACAACAGYCAGAGACGYAGGAY	14317.50
Extension product, F	40	Cy3-AGGGAGTTGGTCTGAGTGACAACAGYCAGAGACGYAGGA	14349.55
F+Y	41	Cy3-AGGGAGTTGGTCTGAGTGACAACAGYCAGAGACGYAGGAY	15254.53
F+dA	41	Cy3-AGGGAGTTGGTCTGAGTGACAACAGYCAGAGACGYAGGAA	14662.76
M+dA	40	TCCTACGTCTCTGACTGTTGTCACTCAGACCAACTCCCTA	12078.75
M+dG	40	TCCTACGTCTCTGACTGTTGTCACTCAGACCAACTCCCTG	12094.75
M+Y	40	TCCTACGTCTCTGACTGTTGTCACTCAGACCAACTCCCTY	12670.52
M-dT+Y	39	TCCTACGTCTCTGACTGTTGTCACTCAGACCAACTCCCY	12366.33
M-dT+Y+dA	40	TCCTACGTCTCTGACTGTTGTCACTCAGACCAACTCCCYA	12679.54
M-dT+Y+dG	40	TCCTACGTCTCTGACTGTTGTCACTCAGACCAACTCCCYG	12695.54
M-dT+Y+Y	40	TCCTACGTCTCTGACTGTTGTCACTCAGACCAACTCCCYY	13271.31
M+dA+dA	41	TCCTACGTCTCTGACTGTTGTCACTCAGACCAACTCCCTAA	12391.96
M+Y+dA	41	TCCTACGTCTCTGACTGTTGTCACTCAGACCAACTCCCTYA	12983.73
incomplete extension product	36	Cy3-AGGGAGTTGGTCTGAGTGACAACAGYCAGAGACGY	13064.71
H1+Y	34	AGGGAGTTGGTCTGATTTTCAGACCAACTCCCTY	11025.51
H1+dA	34	AGGGAGTTGGTCTGATTTTCAGACCAACTCCCTA	10433.74
H1-dT+Y	33	AGGGAGTTGGTCTGATTTTCAGACCAACTCCCY	10721.32
H4-dT	33	Cy3-AGGGAGTTGGTCTGATTTTCAGACCAACTCCC	10753.37
H4-dT+Y	34	Cy3-AGGGAGTTGGTCTGATTTTCAGACCAACTCCCY	11658.35
Degraded H4 (*Pfu*)	25	Cy3-AGGGAGTTGGTCTGATTTTCAGAC	8376.86

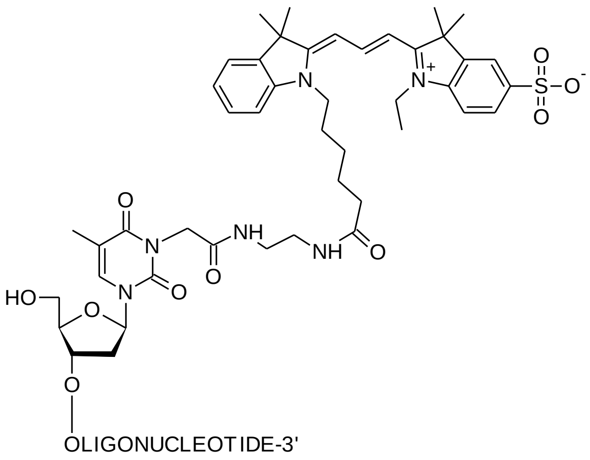

Figure SF8. Structure of Cy3 tag at the primer 5’ end.

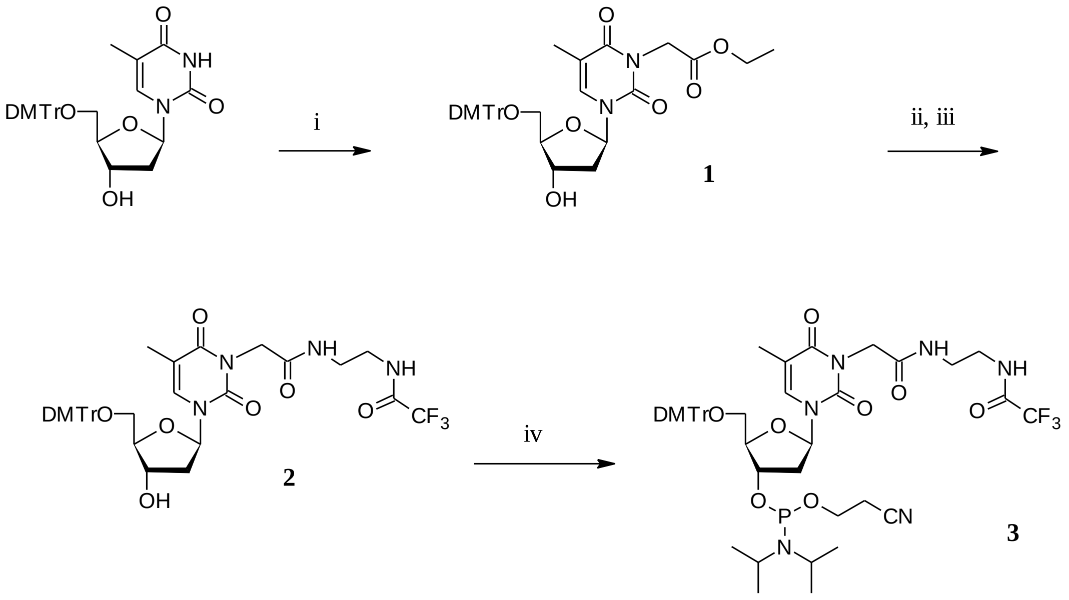

Scheme SS1. Synthesis of dT-aminomodifier phosphoramidite. (i) Ethyl bromoacetate, DBU in MeCN; (ii) Etylene diamine in EtOH, reflux; (iii) Ethyl trifluoroacetate in EtOH; (iv) 2-cyanoethyl-*N*,*N*,*N′*,*N′*-tetraisopropyl-phosphoramidite, tetrazole in MeCN.

5'-O-(4,4'-dimethoxytrityl)-N3-ethoxycarbonylmethylthymidine (1). DBU (2.5 mL, 17 mmol) and ethyl bromoacetate (1.1 mL, 10 mmol) were added to a stirred solution of 5'-O-(4,4'-dimethoxytrityl)-thymidine (1.84 g, 3.4 mmol) in 5 mL MeCN. After 30 min, the reaction mixture was diluted with ethyl acetate (200 mL) and washed with saturated aqueous $NaHCO_3$ (150 mL). The organic layer was dried over anhydrous Na_2SO_4 and evaporated. The product was purified by column chromatography on silica gel (gradient elution from DCM/hexane 1:1 to DCM/EtOH 9:1) to yield 1.98 g (93%) of the title compound. ^{1}H NMR (400 MHz, DMSO-d6) δ 7.64 (2s, 1H, H-6, diastereomers), 7.22-7.40 and 6.89-6.91 (m, 13H, aromatic DMTr), 6.24 (t, *J* = 6.6 Hz, 1H, H-1′), 5.35 (d, *J* = 4.5 Hz, 1H, OH-3′), 4.56 (s, 2H, CH_2 at *N*3), 4.34-4.38 (m, 1H, H-3′), 4.13 (q, *J* = 7.0 Hz, 2H, CH_2 from ethyl), 3.91-3.95 (m, 1H, H-4′), 3.73 and 3.74 (2s, 6H, OCH_3, diastereomers), 3.18-3.28 (m, 2H, H-5′), 2.18-2.32 (m, 2H, H-2′), 1.50 and 1.51 (2s, 3H, CH_3-5, diastereomers), 1.20 (t, *J* = 7.0 Hz, 3H, CH_3 from ethyl); ^{13}C NMR (100 MHz, DMSO-d6) δ 167.7, 162.1, 158.1, 150.0, 144.6, 135.4, 135.2, 134.9, 129.7, 127.8, 127.6, 126.7, 113.2, 108.6, 85.8, 85.7, 84.8, 70.3, 63.6, 60.9, 54.9, 13.9, 12.1; HRMS calcd for $C_{35}H_{38}N_2O_9$ [(M+Na)$^+$]: 653.2470, found: 653.2464.

5'-O-(4,4'-dimethoxytrityl)-N3-(2-trifluoroacetylamino)ethoxycarbonylmethylthymidine (2). Compound **1** (1.0 g, 1.59 mmol) was dissolved in 30% (v/v) ethylenediamine in EtOH (15 mL) and refluxed for 18 hours. Then, the reaction mixture was concentrated and dissolved in DCM (200 mL). The solution was washed with water, dried over anhydrous Na_2SO_4, and evaporated. The residue was dried over P_2O_5 in a desiccator under reduced pressure for 12 hours and then dissolved in EtOH (5 mL). Diisopropylethylamine (1.4 mL, 7.9 mmol) and ethyl trifluoroacetate (0.57 mL, 4.8 mmol) were added to the solution under stirring. After 15 min, the reaction mixture was concentrated *in vacuo* and purified by column chromatography on silica gel (gradient elution from DCM/hexane 1:1 to DCM/EtOH 9:1). Yield 1.02 g (87%). ^{1}H NMR (400 MHz, DMSO-d6) δ 9.37 (t, *J* = 5.0 Hz, 1H, amide $NHCOCF_3$), 8.21 (t, *J* = 5.2 Hz, 1H, amide $NHCOCH_2$), 7.6 (2s, 1H, H-6, diastereomers), 7.22-7.41 and 6.89-6.92 (m, 13H, aromatic DMTr), 6.24 (t, *J* = 7.1 Hz, 1H, H-1′), 5.33 (d, *J* = 5.0 Hz, 1H, OH-3′), 4.40 (s, 2H, CH_2 at *N*3), 4.33-4.38 (m, 1H, H-3′), 3.90-3.93 (m, 1H, H-4′), 3.74 (2s, 6H, OCH_3), 3.19-3.27 (m, 6H, $(CH_2)_2$ and H-5′), 2.15-2.3 (m, 2H, H-2′), 1.48 and 1.49 (2s, 3H, CH_3-5); ^{13}C NMR (100 MHz, DMSO-d6) δ 166.9, 162.3, 158.2, 150.2, 144.6, 135.4, 135.2, 134.5, 129.7, 127.9, 127.7, 126.8, 113.24, 108.6, 85.9, 85.6, 84.7, 70.4, 63.6, 55.0, 45.7; HRMS calcd for $C_{37}H_{39}F_3N_4O_9$ [(M+Na)$^+$]: 763.2561, found: 763.2559.

5'-O-(4,4'-dimethoxytrityl)-N3-(2-trifluoroacetylamino)ethoxycarbonylmethylthymidine-3'-O-[(2-cyanoethyl)-N,N-diisopropylphosphoramidite] (3). 2-cyanoethyl *N*,*N*,*N*′,*N*′-tetraisopropylphosphorodiamidite (0.43 mL, 1.41 mmol) was added under intensive stirring to a solution of **2** (1.0 g, 1.35 mmol), pyridine (0.11 mL, 1.35 mmol), and tetrazole (95 mg, 1.35 mmol) in MeCN. The reaction was allowed to proceed for 1 hour at room temperature. Then, it was diluted with ethyl acetate (150 mL), washed twice with cold saturated aqueous $NaHCO_3$ (150 mL) and then washed with water (150 mL). The organic layer was dried over anhydrous Na_2SO_4 and evaporated to white foam. Yield 1.2 g (95%). ^{1}H NMR (400 MHz, DMSO-d6) δ 9.35-9.39 (m, 1H, amide $NHCOCF_3$), 8.20-8.23 (1H, amide $NHCOCH_2$), 7.62 and 7.63 (4s, 1H, H-6, diastereomers), 7.24-7.42 and 6.88-6.92 (m, 13H, aromatic DMTr), 6.25 and 6.26 (2t, J = 7.0 Hz, 1H, H-1′), 4.54-4.61 (m, 1H, H-3′), 4.39-4.40 (m, 2H, CH_2 at *N*3), 3.97-4.12 (m, 1H, H-4′), 3.74 (2s, 6H, OCH_3), 3.40-3.66 (m, 4H, 2H from *i*-Pr and 2H from CH_2CN), 3.19-3.28 (m, 6H, $(CH_2)_2$ and H-5′), 2.49-2.51 (m, 2H, OCH_2), 2.29-2.45 (m, 2H, H-2′), 1.50 and 1.53 (4s, 3H, CH_3-5), 1.09-1.21 (m, 12H, CH_3 from *i*-Pr); ^{13}C NMR (100 MHz, DMSO-d6) δ 166.8, 162.3, 158.2, 150.2, 144.5, 135.2, 135.0, 134.4, 134.3, 129.7, 127.8, 127.6, 126.8, 118.8, 118.6, 113.2, 108.8, 86.0, 84.7, 84.6,

84.2, 58.3, 58.1, 55.0, 44.4, 42.9, 42.5, 24.2, 22.6; ^{31}P MNR (162 MHz, DMSO-d6) δ 150.4, 150.1; H-C-P; HRMS calcd for $C_{46}H_{56}F_3N_6O_{10}P$ [$(M+Na)^+$]: 963.3640, found: 963.3626.

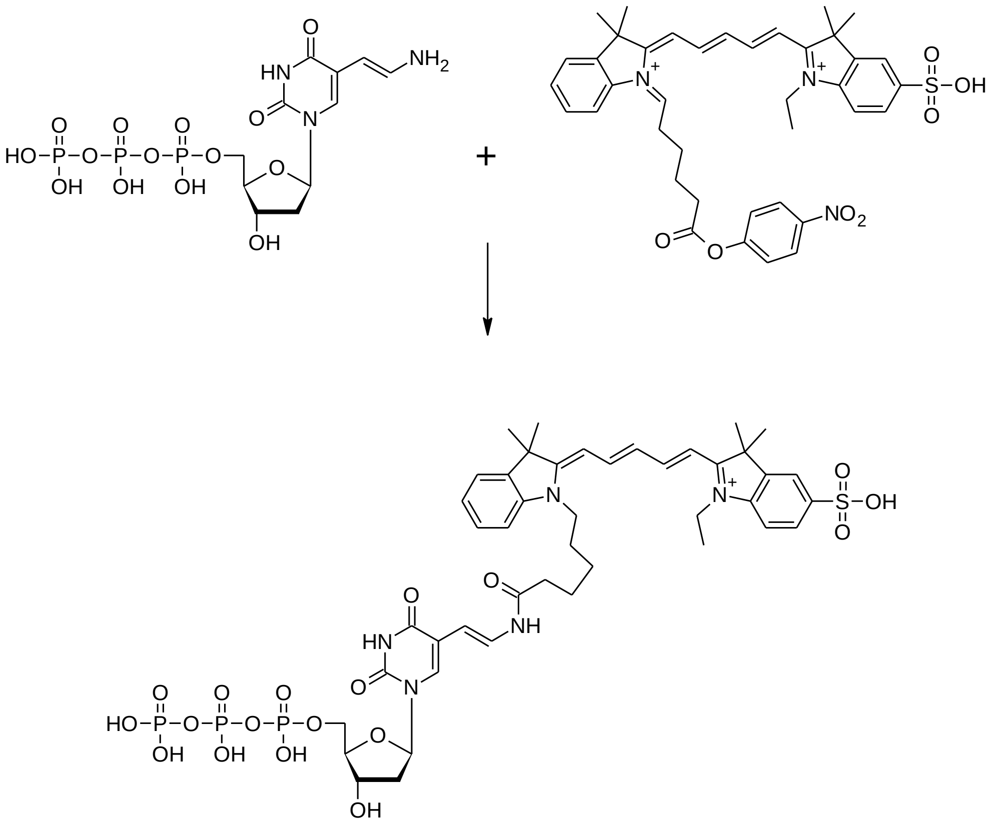

Scheme SS2. Synthesis of non-natural nucleotide Cy5-dUTP (Y).

Cy5-dUTP (Y). Cy5 dye (2 μmol) *p*-nitrophenyl ester in 0.2 mL DMF was added to a cooled (5°C) solution of 5-(3-aminoallyl)-2′-deoxyuridine 5′-triphosphate (1 mg, 1.6 μmol) in 0.2 mL of 0.1 M sodium bicarbonate buffer (pH 8.5). The mixture was stirred for 12 h at 5°C. The unreacted dye was removed by precipitation with 2% $LiClO_4$ in acetone (1.6 mL). Labeled nucleotide was separated by centrifugation and purified on DEAE cellulose (2x8 cm) using a gradient elution with trietylammonium bicarbonate (0.025 to 0.3 M, pH 8.5) in 30% aqueous acetonitrile. The target fractions were applied to a C18 column (2x8 cm), washed with 0.1 M aqueous $NaClO_4$ containing 0.1 M EDTA (pH 8.0) and μQ water, and eluted with 50% acetonitrile in μQ water. The Cy5-dUTP concentration was determined by UV absorbance at 644 nm (ε 210000 $M^{-1}cm^{-1}$). The solution was evaporated to dryness *in vacuo* at 25°C and coevaporated twice with μQ water. Yield 1.2 mg (64%). ^1H NMR (400 MHz, D_2O) δ 7.82-7.99, 7.73-7.75, and 7.19-7.47 (m, 10H, dye β,β'-CH, dye aromatic, and H-6), 6.08-6.48 (m, 6H, dye γ-CH, dye H-1, dye H-2, H-1', and dye α,α'-CH), 3.71-4.23 (m, 9H, H-3', H-4', H-5', CH_2 from ethyl, CH_2NHCO, and CH_2-10 from amide linker), 2.22-2.32 (m, 4H, H-2', and CH_2-6 from amide linker), 1.59 (2s, 12H, CH_3), 1.62-1.85 and 1.32-1.42 (m, 6H, $(CH_2)_3$ from amide linker), 1.28 (t, *J* 7.0 Hz, 3H, CH_3 from ethyl). ^{31}P NMR (162 MHz, D_2O) δ -21.69 (t, βP), -10.95 (d, αP), -7.69 (d, γP). MS (MALDI-TOF) calcd. for $C_{45}H_{59}N_5O_{18}P_3S$ [M^+]: 1078.6, found: 1082.3.
